# ROS scavenging and ion homeostasis is required for the adaptation of halophyte *Karelinia caspia* to high salinity

**DOI:** 10.3389/fpls.2022.979956

**Published:** 2022-10-03

**Authors:** Cui Li, Luis A.J. Mur, Qinghai Wang, Xincun Hou, Chunqiao Zhao, Zhimin Chen, Juying Wu, Qiang Guo

**Affiliations:** ^1^Institute of Grassland, Flowers and Ecology, Beijing Academy of Agriculture and Forestry Sciences, Beijing, China; ^2^Institute of Biological, Environmental and Rural Sciences, Aberystwyth University, Aberystwyth, United Kingdom; ^3^College of Software, Shanxi Agricultural University, Taigu, China; ^4^College of Horticulture and Landscape, Tianjin Agricultural University, Tianjin, China

**Keywords:** halophyte, *Karelinia caspia*, ion homeostasis, antioxidant activity, photosynthesis, salt stress

## Abstract

The halophyte *Karelinia caspia* has not only fodder and medical value but also can remediate saline-alkali soils. Our previous study showed that salt-secreting by salt glands is one of main adaptive strategies of *K. caspia* under high salinity. However, ROS scavenging, ion homeostasis, and photosynthetic characteristics responses to high salinity remain unclear in *K. caspia*. Here, physio-biochemical responses and gene expression associated with ROS scavenging and ions transport were tested in *K. caspia* subjected to 100–400 mM NaCl for 7 days. Results showed that both antioxidant enzymes (SOD, APX) activities and non-enzymatic antioxidants (chlorogenic acid, α-tocopherol, flavonoids, polyamines) contents were significantly enhanced, accompanied by up-regulating the related enzyme and non-enzymatic antioxidant synthesis gene (*KcCu/Zn-SOD*, *KcAPX6*, *KcHCT, KcHPT1*, *Kcγ-TMT, KcF3H*, *KcSAMS* and *KcSMS*) expression with increasing concentrations of NaCl. These responses are beneficial for removing excess ROS to maintain a stable level of H_2_O_2_ and O_2_^−^ without lipid peroxidation in the *K. caspia* response to high salt. Meanwhile, up-regulating expression of *KcSOS1/2/3, KcNHX1,* and *KcAVP* was linked to Na^+^ compartmentalization into vacuoles or excretion through salt glands in *K. caspia*. Notably, salt can improve the function of PSII that facilitate net photosynthetic rates, which is helpful to growing normally in high saline. Overall, the findings suggested that ROS scavenging systems and Na^+^/K^+^ transport synergistically contributed to redox equilibrium, ion homeostasis, and the enhancement of PSII function, thereby conferring high salt tolerance.

## Introduction

Soil salinization has become one of the most severe global environmental problems. It covers almost 7% of agricultural land on earth and predictions suggest that the continuous expansion of salinization may lead to a loss of 50% agricultural land by 2050 (Wang et al., 2003). Most of crops are glycophytes, and have a low tolerance threshold for salinity (Ismail and Horie, 2017). However, halophytes are evolved the adaptive mechanism to complete life cycle in high salt environments more than 200 mM NaCl (Flowers and Colmer, 2008). Therefore, underlying the mechanisms of halophytes to adapt to salt stress could be important in breeding salt-tolerance crops (Guo et al., 2020).

Unlike crops, halophytes utilize not only organic osmotic substances but also inorganic osmolytes for osmotic adjustment (Sanadhya et al., 2015). Zeng et al. (2015) reported that inorganic ions may play more important roles than organic compounds in NaCl-induced osmotic adjustment in *Halostachys caspica*. The contribution of Na^+^ to osmotic potential was up to 60% in halophyte *Solanum chilense* (Gharbi et al., 2017), which is mainly achieved by sequestration of Na^+^ into the vacuole. Tonoplast Na^+^/H^+^ antiporters *NHX* and *AVP* mediated Na^+^ movement in the vacuole (Liu et al., 2012; Toranj et al., 2020), Meanwhile, halophytes secreted excess Na^+^ by salt glands, and the rate of salt secretion increased with the increasing salinity (Sanadhya et al., 2015). In *K. caspia, KcSOS1* silenced-lines disrupted the Na^+^ transport, then decreased Na^+^ secretion rates (Guo et al., 2020). These findings imply that *SOS1* mediated the Na^+^ efflux in leaves, and may be the main regulator in salt secretion to maintain Na^+^ homeostasis (Shi et al., 2002). Thus, regionalization of excess Na^+^ into vacuoles or excretion reduced the concentration of Na^+^ in the cytoplasm and maintained the balance of ions by regulating ion channel genes. Controlled uptake and compartmentalization of Na^+^ enhanced the tolerance of halophytes to salinity (Flowers and Colmer, 2008). Besides, salinity induces the production of excess reactive oxygen species (ROS), including H_2_O_2_ and O_2_^−^ in plants (Hernández et al., 2001). SOD has been considered the first defense against oxidative stress by dismutating O_2_^−^ to H_2_O_2,_ and antioxidant enzymes like CAT, and POD catalyzed the H_2_O_2_ into water and oxygen, to reduce damage by ROS (Bose et al., 2014). Further research demonstrated that overexpression of antioxidant enzyme genes such as *CAT* and *POD* regulated H_2_O_2_ homeostasis and improved the tolerance to salt stress (Zhou et al., 2018; Jin et al., 2019). Apart from the role of antioxidant enzymes in ROS scavenging, secondary metabolities such as chlorogenic acid, flavonoids, tocopherols, and polyamines play critical roles in the antioxidant mechanism (Guo et al., 2022). For example, Jin and Daniell (2014) reported that the overexpression of *γ-TMT* increased the content of α-tocopherol, then decreased the production of ROS, thus improving the tolerance of tobacco to salinity. Chlorogenic acid accumulation in *Lonicera japonica* suppressed the ROS content under salt treatments (Yan et al., 2017). Similarly, flavonoids were increased when exposed to salinity, and the accumulation of flavonoids effectively scavenged the hydroxyl radicals induced by salinity (Ismail et al., 2016). Above findings indicated that halophytes resist salt stress by regulating ionic homeostasis as well as ROS. However, most of previous studies only focused on ROS homeostasis or ion homeostasis (Al-Shamsi et al., 2020; Tran et al., 2020). The mechanism by which ROS and ion homeostasis combined to withstand salt stress remains unclear. In this study, we comprehensively explored the mechanisms of salt tolerance from ROS homeostasis, ion homeostasis, as well as osmotic adjustment. This was important to reveal the tolerance mechanism of halophytes to salinity.

*Karelinia caspia* is a recretohalophyte, perennial herb Asteraceae mainly distributed in semi-desert areas and desert grassland in Northwestern China, it is a pioneer species for improving saline-alkali and desertified soil, and it is also an important forage species for livestock in desert grassland (Wang et al., 2013). Previous studies have shown that *KcSOS1* mediates the excretion of Na^+^ by salt glands (Guo et al., 2020), and *NHX1* mediates the Na^+^ regionalization into vacuoles (Liu et al., 2012), thereby maintaining the ion homeostasis. Other adaptive mechanisms such as plant growth, osmotic adjustment, ionic homeostasis, ROS regulation of *K. caspia* to high saline stress need to be explored. Herein, we investigated the adaptation mechanisms of *K. caspia* by measuring plant growth, chlorophyll fluorescence, ion distribution, osmoregulation, enzymatic and non-enzymatic antioxidant components, and releted gene expression. This finding would provide valuable information for the remediation of *K. caspia* used in saline-alkali soil and the development of salt-resistant crop breeding.

## Materials and methods

### Plant material and experiment conditions

Seeds of *K. caspia* were collected from the experimental field at the College of Grassland and Environment Sciences of Xinjiang Agricultural University. Seeds were germinated in a 2:1 mixture of peat and sand under ambient conditions [temperature: 25°C/18°C (day/night), photoperiod: 16 h/8 h (day/night), relative humidity: 60%, photosynthetically active radiation: 300 μmol m^−2^ s^−1^]. They were watered with Hoagland nutrient solution (Guo et al., 2017). After 6 weeks, the seedlings were transplanted to an opaque plastic culture tank (10*20 cm; hight*diameter) containing nutrient solutions. After 1 week of acclimation, seedlings were treated with solutions supplemented with 0, 100, 200, 300, and 400 mM NaCl. The salinity of the nutrient solutions was increased gradually by adding 100 mM NaCl per day until the designed concentrations were attained. Each treatment was repeated three times, and every replicate had two seedlings. Solutions were renewed every 3 days to maintain a stable concentration of NaCl. The duration of NaCl treatment was 7 days.

### Measurement of growth parameters

Plant growth parameters such as relative growth rate (RGR), relative water content (RWC), and salinity tolerance index (STI) were measured in the experiment. The relative growth rate was calculated using the formula (Martínez et al., 2005).; W_f_ is the final dry weight, and W_i_ is the initial dry weights, Δ*t* is the time of treatment. Relative water content was calculated using the formula (Aymen et al., 2016). Salinity tolerance index was calculated using the formula (Panda et al., 2019).


$$\mathrm{STI}\left(\%\right)=\frac{Dry\;biomassoftreatedplant}{Dry\;biomassofcontrol}\times 100.$$

$$\mathrm{RGR}=\frac{\ln Wf-\ln Wi}{\Delta t}$$

### Measurement of photosynthetic pigments, chlorophyll fluorescence and gas exchange parameters

Chlorophylls (Chl a and b) and total chlorophyll content were measured according to the method of Dere et al. (1998). The absorbance was measured at 470 nm, 645 nm, and 663 nm for chlorophylls calculation.

Chlorophyll fluorescence parameters were determined by the Handy PEA analyzer (Hansatech, United Kingdom). Firstly, leaf was covered by a leaf clip for 30 min for dark adaption, then opened the leaf clip and measured chlorophyll fluorescence by exposing the leaf to a 2 s saturating light pulse of 3,500 μmol photons m^−2^ s^−1^. The parameters were calculated according to the protocol provided by the manufacturer. Thirty replicates were measured for every treatment.

Gas exchange in leaves was measured using a CIRAS-3 portable photosynthesis system (PP System, United States). The reference CO_2_ concentration was maintained at 400 μmol·mol^−1^, and photon flux density was set at 300 μmol m^−2^ s^−1^. Gas exchange measurements were taken in the artificial climate chamber; five leaves were measured for every treatment.

### Determination of cations concentration

Mineral ion content was measured according to the method of Rangani et al. (2018). Firstly, fresh leaves were dried at 60°C for 72 h and then ground into powder. The samples were dissolved in HNO_3_ and HClO_4_ mixture (4:1) and digested using microwave acid digestion. The extraction was filtered with a 0.45 μm filter, and then it was used to measure ions by inductively coupled plasma-optical emission spectrometry (ICP-OES; Agilent, United States).

### Measurement of soluble sugars and proline

The content of soluble sugars was measured by the anthrone method (Watanabe et al., 2000). The absorption was measured at 620 nm. Proline content was measured according to the method of Bates et al. (1973). Fresh leaves were homogenized with 3% sulfosalicylic acid and then centrifuged at 14,000 g for 10 min. The mixture consisting of leaf extract, glacial acetic acid, and acid-ninhydrin was incubated at 100°C for 30 min, then added toluene and aspirated the upper solution. The absorption was measured at 520 nm.

### Measurement of osmotic potential and relative contribution of inorganic and organic solutions

Osmotic potential (Ψs) and relative contribution of organic and inorganic solutes for osmotic adjustment were measured by the method of Silveira et al. (2009). Fresh leaves were homogenized with pestle mortar and centrifuged at 10,000 g for 10 min at 4°C. Then the supernatant was used to detect osmolality using a vapor pressure osmometer (Gonotec, German). The osmotic potential was calculated with the van’t Hoff equation Ψs=−nRT , where *n* is the osmolarity of solutions, *R* is the gas constant, and *T* is the absolute temperature. The relative contribution (RC) of organic and inorganic solutes to the osmotic potential was estimated as % the osmolality (Silva et al., 2010).


$$\mathrm{RC}=\frac{\mathrm{Solute concentration}\;\left(\mathrm{mmol}/\mathrm{kg}\;\mathrm{water tissue}\right)}{\mathrm{Osmolality}\;\left(\mathrm{mmol}/\mathrm{kg}\;\mathrm{solvent}\right)\;}.$$

### Determination of lipid peroxidation, antioxidant enzyme and non-enzymatic antioxidants

To indicate lipid peroxidation, malondialdehyde (MDA) levels were measured according to the method of Cakmak and Marschner (1992) with slight modification. Fresh leaves (0.5 g) was ground into homogenization with 0.5% trichloroacetic acid (TCA), then centrifuged the homogenous at 15,000 g at 4°C for 10 min. The supernatant was collected and 0.6% thiobarbiituric acid (v/v) was added and transferred to new tube. The tubes were heated at 95°C for 15 min, and quickly cooled in an ice bath. The absorbance at 450 nm, 532 nm, and 600 nm was measured by spectrophotometer (Persee, China).

### Determination of H_2_O_2_ and O_2_^−^

The level of H_2_O_2_ in leaves was measured by examining the absorbance of the titanium peroxide complex at 415 nm according to the method of Hu et al. (2020). Fresh leaves (0.1 g) were ground into a homogenous slurry with 1 mL acetone, then centrifuged at 8, 000 g at 4°C for 10 min. The supernatant was collected (v/v) and 5% titanium sulfate solution and ammonia water added. This was centrifuged at 4000 g, at 25°C for 10 min and pellet collected. The pellets were dissolved in 2 mM sulfuric acid and the absorbance measured at 415 nm. Superoxide can react with hydroxylamine hydrochloride to form red azo compounds, and and so O_2_- levels were calculated by measuring the absorbance at 530 nm as described by Yang et al. (2011).

Nitroblue tetrazolium (NBT) and 3,3-diaminobenzidine (DAB) staining was used to detect O_2_^-^ and H_2_O_2_ *in situ*. NBT staining was carried out by the method of Fryer et al. (2002). The third leaf was dipped in NBT solution, under vacuum for 20 min, and then left to stand at room temperature for 1 h. After this, the leaf is decolorized in 95% ethanol solution. To detect H_2_O_2_, the third expanded leaf was used and wholly immersed in DAB solution, incubated for 8 h, then placed in 95% ethanol to decolorize (Thordal-Christensen et al., 2002).

### Measurement of antioxidative enzyme activities and non-enzymatic antioxidants

To determine antioxidant enzyme activities and the levels of non-enzymatic antioxidants, fresh leaves (0.5 g) were ground into powder with liquid nitrogen and mortar and pestle. The activity of superoxide dismutase (SOD, EC 1.15.1.1), peroxidases (POD, EC 1.11.1.7) was measured using the method described by Guo et al., (2017). Ascorbate peroxidase (APX, EC 1.11.1.11) was measured by the decreased absorbance at 290 nm for 1 min (Nakano and Asada, 1981). Glutathione reductase (GR, EC 1.6.4.2) activity was calculated by measuring the absorbance of NADPH at 340 nm (Foyer et al., 1991). The reduced ascorbate (AsA) content and total ascorbate (AsA + DHA) were measured following the method of Gillespie and Ainsworth, (2007). Glutathione content including reduced (GSH) and total glutathione (GSH + GSSG) was measured according to Anderson (1985). Flavonoid contents were measured by the method of Jia et al., (1999).

α-tocopherol content was measured following the method of Zhang et al., (2020). Fresh leaves (0.5 g) were homogenized with a solution containing methanol and chloroform (2:1) and the extract was filtered with a 0.22 μm filter. High performance liquid chromatography (HPLC) was used with a C_18_ column (250*4.6 nm, 5 μm). The mobile phase consisted of 95% methanol and 5% isopropanol, and α-tocopherol was detected at 280 nm. Concentrations were calculated by a standard curve of α-tocopherol.

Chlorogenic acid content was assayed according to the method (Yan et al., 2016). Dry leaf powder (1 g) was homogenized in 30 mL 70% methanol and ultrasonic extraction for 30 min. The extraction was centrifuged for 10 min at 10,000 g. The assay was performed in high performance liquid chromatography (HPLC) with a C_18_ column (250*4.6 nm, 5 μm). The mobile phase was acetonitrile (13%) and 0.3% phosphoric acid (87%). Chlorogenic acids were detected at 327 nm, and their concentrations were determined using a standard curve of known concentrations of chlorogenic acid.

Fresh leaves were collected for the measurement of polyamines (spermine and spermidine). Fresh leaves (0.5 g) was grounded into power, homogenized with 1% perchloric acid and incubated for 1 h, centrifuged at 15000 g for 20 min. Then 200 μL saturated Na_2_CO_3_ and 400 μL dansyl chloride was added to the supernatant, and incubated overnight. Finally, the organic phase was extracted into 500 μL toluene and filtered with 0.22 μm filter. Spermine and spermidine levels were measured by HPLC as described by Marcé et al. (1995).

### Gene expression assay

After 7 days of salinity treatments, leaves was collected for RNA extractions and gene expression assessments. RNA was extracted using the plant RNA Extraction Kit (TaKaRa, 9,769), then 1 μg RNA was reverse-transcribed to cDNA using Prime Script RT reagent Kit (TaKaRa, RR820A). qRT-PCR was carried out by CFX Connect real-time system (BioRad, United States) using a SYBR PCR mix (TaKaRa, RR420A). Thermal cycling conditions were 95°C for 30s, followed by 40 cycles of 95°C for 5 s, 60°C for 30s. The primer sequences of oftarget genes are listed in Supplementary Table S1. Gene expression was calculated using the formula 2^-ΔΔCT^ and *KcActin* was used as the reference gene (Guo et al., 2020).

### Statistical analyses

Data were presented as mean ± standard deviation (SD). One-way ANOVA was performed with Duncan’s multiple range test (*p* ≤ 0.05) using software SPSS22. There were 30 replicates for chlorophyll fluorescence parameters, five replicates for gas exchange parameters, and three replicates for all the other parameters. The correlation matrix was performed using Origin 2021.

## Results

### Effects of salinity on plant growth parameters, chlorophyll fluorescence, and photosynthetic efficiency

**Figure 1:**
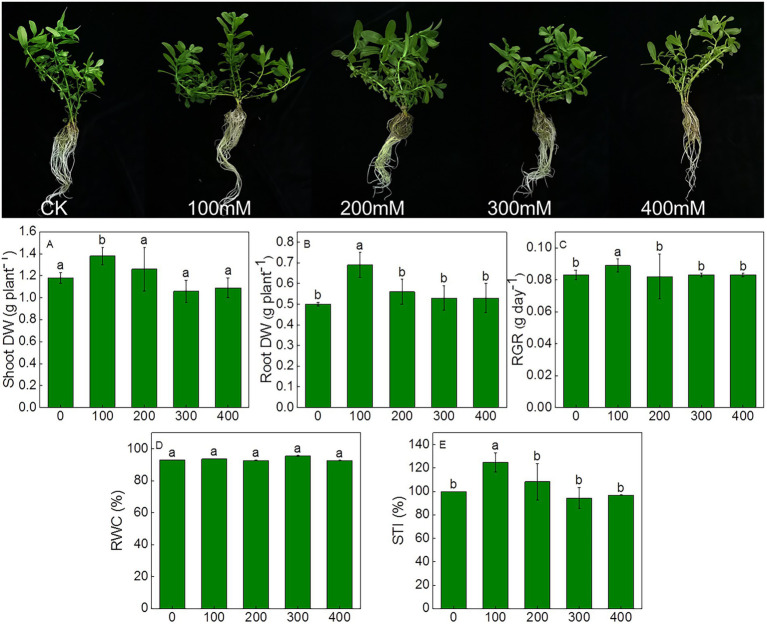
Morphological characteristics and changes in growth parameters of *Karelinia caspia* after 7 days of NaCl (mM) treatment. Different letters indicate significant difference at *p* < 0.05.

The effects of salt stress on the growth, chlorophyll fluorescence and photosynthetic efficiency were determined in *K. caspia*. No salt toxicity symptoms were observed in *K. caspia* after 7 days of NaCl treatment (Figure 1). In *K. caspia*, there was significant increase in shoot and root dry weight in 100 mM NaCl treatment. Similarly, the relative growth rate and salinity tolerance index increased by 7.2 and 24.76% under 100 mM NaCl, respectively. Both RGR and STI showed no significant difference in 200 to 400 mM NaCl treatments compared with controls. RWC was maintained at the same level among all treatments (Figure 1).

Compared to controls, the levels of the photosynthetic pigments such as Chla, Chlb, total chlorophyll, and Chl a/b ratio exhibited no significant differences (Table 1). To assess the effects of salinity on the photosynthetic function, chlorophyll fluorescence was measured using Handy PEA analyzer. The derived parameters provided information concerning the structure and function of PSII. The maximum quantum yield of PSII (F_v_/F_m_) increased significantly in treatments from 200 to 400 mM NaCl. There was no significant difference in ABS/CS_m_ under salinity compared with control. TR_0_/CS_m_ increased significantly under 400 mM NaCl treatment, and ET_0_/CS_m_ increased significantly under the 300 and 400 mM NaCl treatments. The increase of ET_0_/CS_m_ and RE_0_/CS_m_ suggested elevated electron transport and increased efficiency of PSI. As compared to control, DI_0_/CS_m_ decreased in NaCl treatments, especially in 300 and 400 mM NaCl treatment, decreasing by 21.9 and 32.4% compared with control. There was no significant difference in PI_abs_ under NaCl treatment from 100 to 300 mM compared with control, but it increased significantly under 400 mM NaCl treatment (Table 2).

**Table 1:** The level of photosynthetic pigments responses to salinity in *Karelinia caspia*.

NaCl treatment (mmol L^−1^)	Chla (mg g^−1^FW)	Chlb (mg g^−1^FW)	Chl(a + b) (mg g^−1^FW)	Chl a/b
0	0.21 ± 0.02a	0.13 ± 0.01a	0.34 ± 0.03a	1.65 ± 0.12a
100	0.27 ± 0.01a	0.15 ± 0.00a	0.42 ± 0.01a	1.70 ± 0.08a
200	0.22 ± 0.01a	0.13 ± 0.02a	0.35 ± 0.02a	1.79 ± 0.09a
300	0.26 ± 0.05a	0.14 ± 0.07a	0.40 ± 0.07a	1.80 ± 0.02a
400	0.21 ± 0.00a	0.12 ± 0.01a	0.33 ± 0.01a	1.79 ± 0.07a

Different letters indicate significant difference at *p* < 0.05.

**Table 2:** The changes of Chlorophyll fluorescence parameters in response to salinity in *Karelinia caspia.*

NaCl treatment (mmol L^−1^)	Chlorophyll fluorescence parameters					
	F_v_/F_m_	PI abs	Abs/CS_m_	TRo/CS_m_	ETo/CS_m_	DIo/CS_m_
0	0.71 ± 0.01c	0.25 ± 0.07b	1261.47 ± 49.36a	882.05 ± 43.89a	130.77 ± 18.50c	363.07 ± 22.84a
100	0.74 ± 0.01bc	0.27 ± 0.05b	1236.20 ± 74.99a	941.91 ± 43.73a	147.80 ± 15.87bc	334.57 ± 19.08a
200	0.75 ± 0.01b	0.45 ± 0.09b	1134.87 ± 57.50a	851.13 ± 42.13a	149.80 ± 18.70bc	326.60 ± 26.35a
300	0.76 ± 0.01b	0.39 ± 0.08b	1352.60 ± 42.14a	939.57 ± 23.00ab	192.50 ± 17.63b	283.73 ± 21.67b
400	0.80 ± 0.01a	0.95 ± 0.13a	1184.83 ± 36.30a	1018.03 ± 27.07b	274.83 ± 21.49a	245.27 ± 15.61b

Different letters indicate significant difference at *p* < 0.05.

Net photosynthetic rate (Pn) was significantly higher than the control under salt treatments, and they increased by 1.6, 1.9, 1.4, and 0.6 fold, respectively, from 100 to 400 mM NaCl (Table 3). Similarly, water use efficiency (WUE) was also increased significantly in saline treatments compared with control, and it was 3.6, 5.6, 6.4, and 3.9 fold that of the control, respectively, under salt treatments. However, stomatal conductance (gs) and transpiration (E) were all decreased as compared to the control, especially in 400 mM NaCl, stomatal conductance and transpiration decreased by 75.7 and 58.2%, respectively, (Table 3).

**Table 3:** Effects of different salinity on photosynthetic parameters in *Karelinia caspia*.

NaCl treatment (mmol L^−1^)	Pn (μmol m^−2^ s^−1^)	gs (mmol m^−2^ s^−1^)	E (mmol m^−2^ s^−1^)	WUE
0	1.04 ± 0.07d	149.20 ± 6.98a	3.14 ± 0.12a	0.32 ± 0.02c
100	2.60 ± 0.30a	69.00 ± 4.70b	2.29 ± 0.12b	1.16 ± 0.11b
200	3.0 ± 0.12a	47.80 ± 1.39c	1.67 ± 0.06c	1.80 ± 0.09a
300	2.48 ± 0.08b	38.40 ± 3.75c	1.27 ± 0.12d	2.04 ± 0.22a
400	1.64 ± 0.09c	36.20 ± 2.60c	1.31 ± 0.08d	1.26 ± 0.07b

Different letters indicate significant difference at *p* < 0.05.

### Ion accumulation and distribution of *Karelinia Caspia* in response to salinity

**Figure 2:**
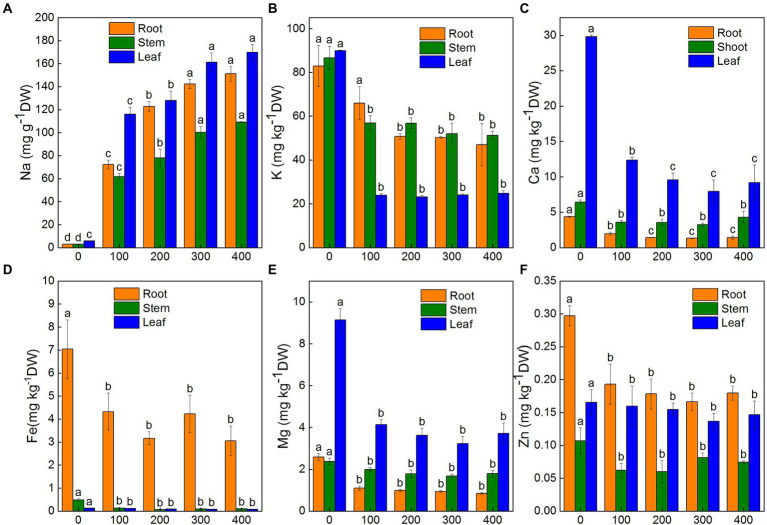
Effects of NaCl treatment (100, 200, 300, and 400 mM) on ions content in roots, stems and leaves of *Karelinia caspia.* **(A)** Na^+^, **(B)** K^+^, **(C)** Ca^2+^, **(D)** Fe^2+^, **(E)** Mg^2+^, **(F)** Zn^2+^. Different letters indicate significant difference at *p* < 0.05

**Figure 3:**
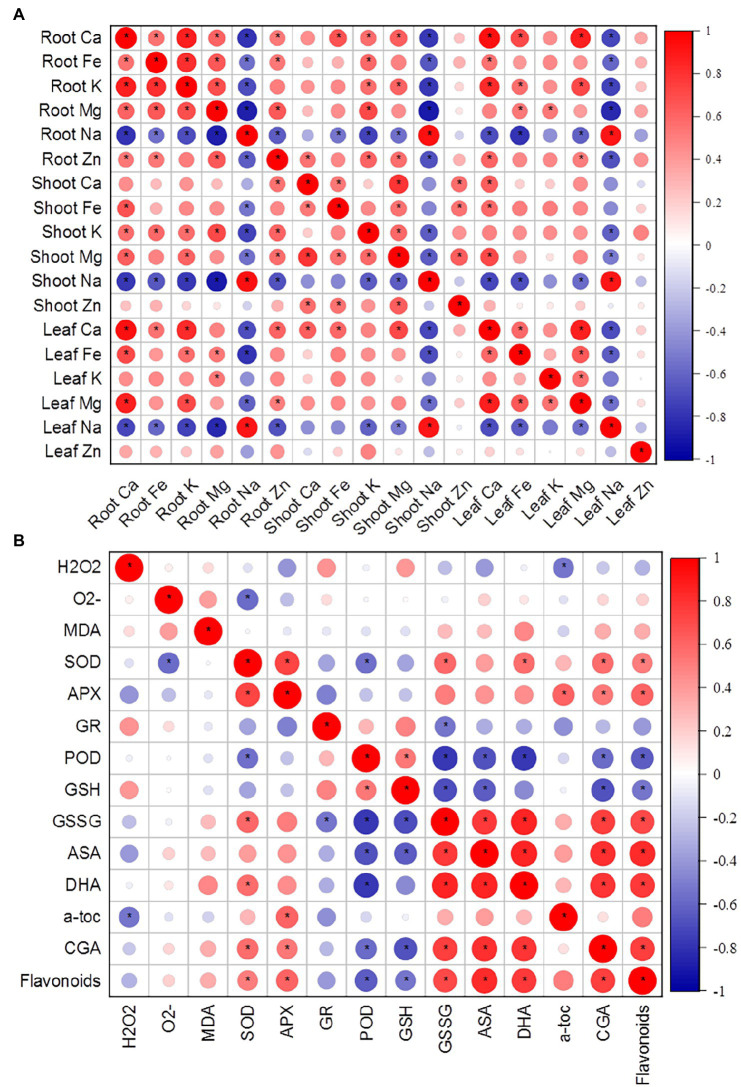
**(A)** Correlation matrix of various ions in roots, shoots, and leaves of *K. caspia*. **(B)** Correlation matrix between antioxidative components (antioxidant enzymes, non-enzymatic antioxidants) and stress indicators (O_2_^−^, H_2_O_2_, MDA). Asterisks indicate significant difference at *p* < 0.05.

To understand how salinity effected the accumulation and distribution of nutrients in plants, K^+^, Na^+^, Ca^2+^, Mg^2+^, Zn^2+^, and Fe^2+^ contents in roots, shoots, and leaves of *K. caspia* were measured after 7 days. The results showed that the content of Na^+^ in roots, shoots, and leaves were significantly and positively correlated with NaCl treatment concentration. Na^+^ content increased significantly in roots, shoots, and leaves compared to the control. With the 400 mM NaCl treatment, Na^+^ content was 48.2, 36.9, and 29.3 times that of the control in roots, shoots, and leaves of *K. caspia,* respectively (Figure 2A). However, the contents of K^+^, Ca^2+^, Mg^2+^, Zn^2+^, and Fe^2+^ were all decreased in roots, shoots, and leaves compared to the control except Zn^2+^ in leaves (Figures 2B–E). The content of Zn^2+^ in NaCl treatments was maintained at the same level as control (Figure 2F). Although ions content decreased compared with control, the content of K^+^, Ca^2+^, Mg^2+^, Zn^2+^, and Fe^2+^ had no significant difference among the treatments except in the root content of K^+^ and Ca^2+^ with 100 mM NaCl treatment. The K^+^/Na^+^ ratio was also maintained at the same level among all NaCl treatments. Correlation analysis showed that the content of Na^+^ in leaves was positively correlated with Na^+^ in roots and stems, but negatively correlated with Ca^2+^, Fe^2+^ and Mg^2+^. Similarly, the content of Na^+^ in roots was negatively correlated with Ca^2+^, Fe^2+^, K^+^, and Mg^2+^ (Figure 3A). These results indicated that *K. caspia* accumulated a large amount of Na^+^, and meanwhile maintained the homeostasis of other ions under salt treatment.

### Relative contributions of organic and inorganic solutes to osmotic adjustment

**Figure 4:**
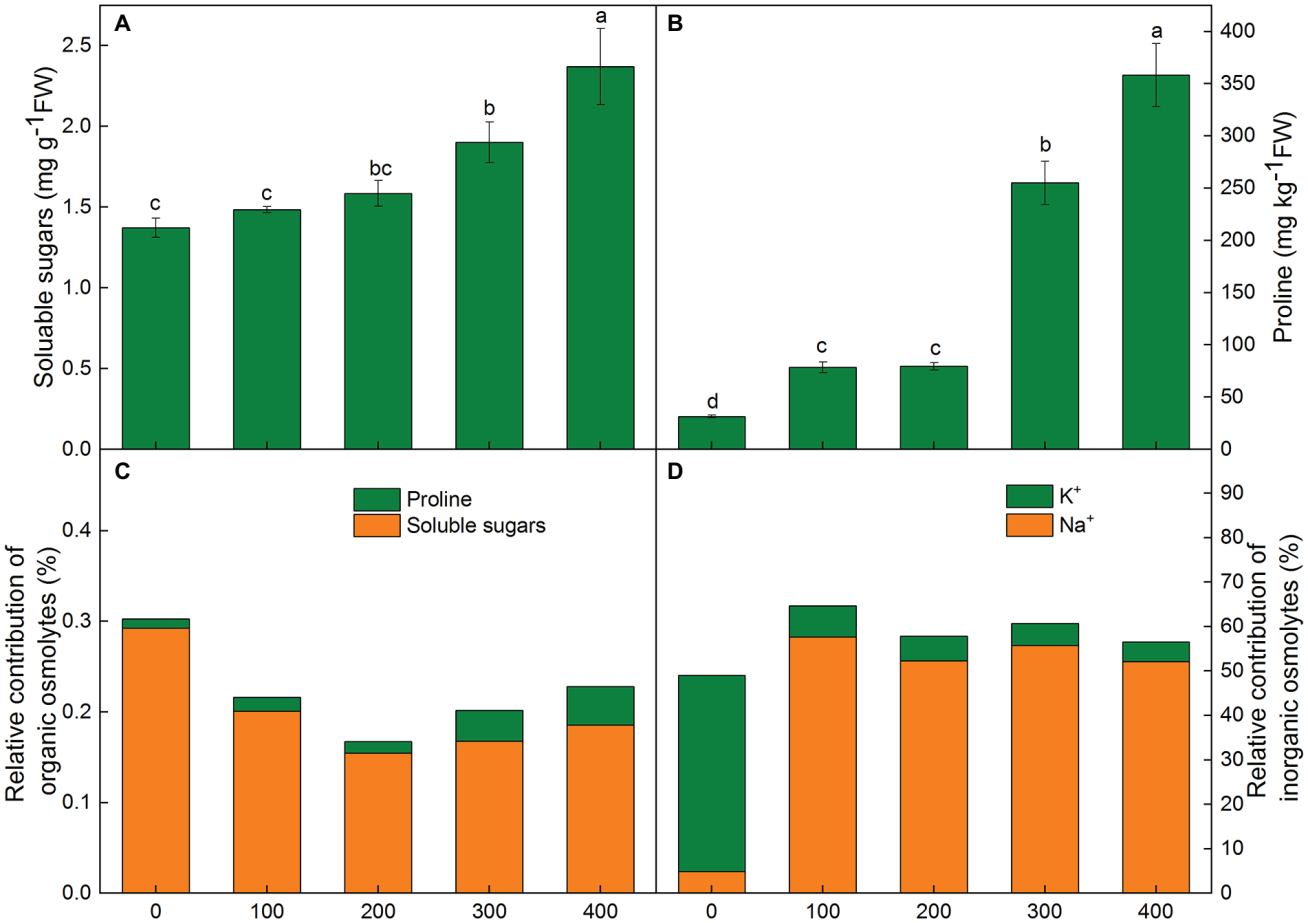
Changes of soluble sugars**(A)**, proline **(B)**, relative contribution of organic **(C)** and inorganic **(D)** solutes for osmotic adjustment in *Karelinia caspia* under salinity (mM NaCl) treatment (100, 200, 300, and 400 mM). Different letters indicate significant difference at *p* < 0.05.

In *K. caspia*, the soluble sugars increased progressively with NaCl treatments. There were significant differences with 300 and 400 mM NaCl treatments as compared to control (Figure 4A). In *K. caspia,* there was a significant increase under all treatments compared with control. The level of proline increased by 1.5, 1.5, 7.1, and 10.4 fold, respectively, in 100, 200, 300, and 400 mM NaCl treatments as compared to control (Figure 4B).

In *K. caspia*, the osmotic potential of leaves decreased with the increasing salinity, and the values of osmotic potential were − 0.97, −1.48, −2.05, −2.51, and − 2.74 MPa, respectively, in control and NaCl treatments from 100 to 400 mM. Our results suggested that the contribution of organic solutes in control was higher than that in NaCl treatments. However, the contributions of inorganic solutes were higher in NaCl treated plants than in control plants. Amongst the organic osmolytes, soluble sugars contributed 0.15–0.29% to osmotic adjustment, and proline had a very negligible contribution (0.01–0.04%) (Figure 4C). Among inorganic osmolytes, the contribution of K^+^ towards osmotic adjustment in control was 44.2%, and the contribution of Na^+^ was only 4.8% in control. However, relative contributions of Na^+^ to osmotic adjustment were 52.3 to 57.6% under salt treatments, and the contribution of K^+^ was 4.4 to 7.0% (Figure 4D). These results showed that Na^+^ acted as the main osmotic solute for osmotic regulation under salt stress.

### Effects of salinity on H_2_O_2_ accumulation, lipid peroxidation, antioxidant enzyme activity and non-enzymatic antioxidants

**Figure 5:**
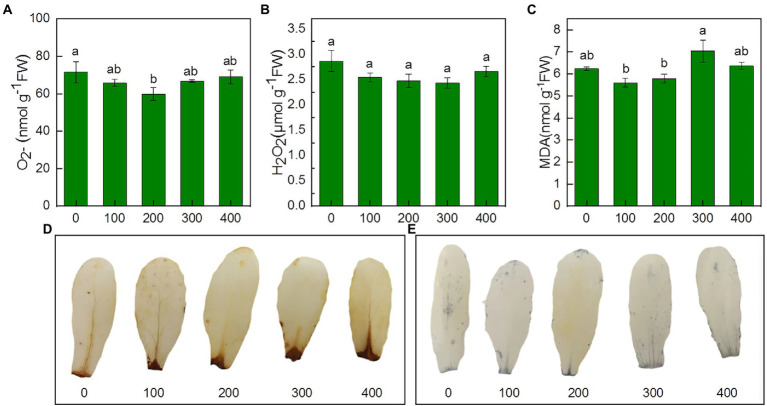
Effects of NaCl treatment (100, 200, 300, and 400 mM) on O_2_^−^、H_2_O_2_ and MDA of *Karelinia caspia.* **(A)** O_2_^−^, **(B)** H_2_O_2_, **(C)** MDA, **(D)** DAB staining for H_2_O_2_, **(E)** NBT staining for O_2_^−^ Different letters indicate significant difference at *p* < 0.05.

To investigate salinity-induced oxidative stress, O_2_^−^, H_2_O_2_, MDA, antioxidant enzyme activity, and content of non-enzymatic antioxidants were examined under salinity. Our results showed that the content of O_2_^−^, H_2_O_2_, and MDA decreased under salinity, but no significant differences were observed between the NaCl treatments and the control (Figures 5A–C). Under salinity, NBT staining and DAB staining have no significant difference with control (Figures 5D–E).

**Figure 6:**
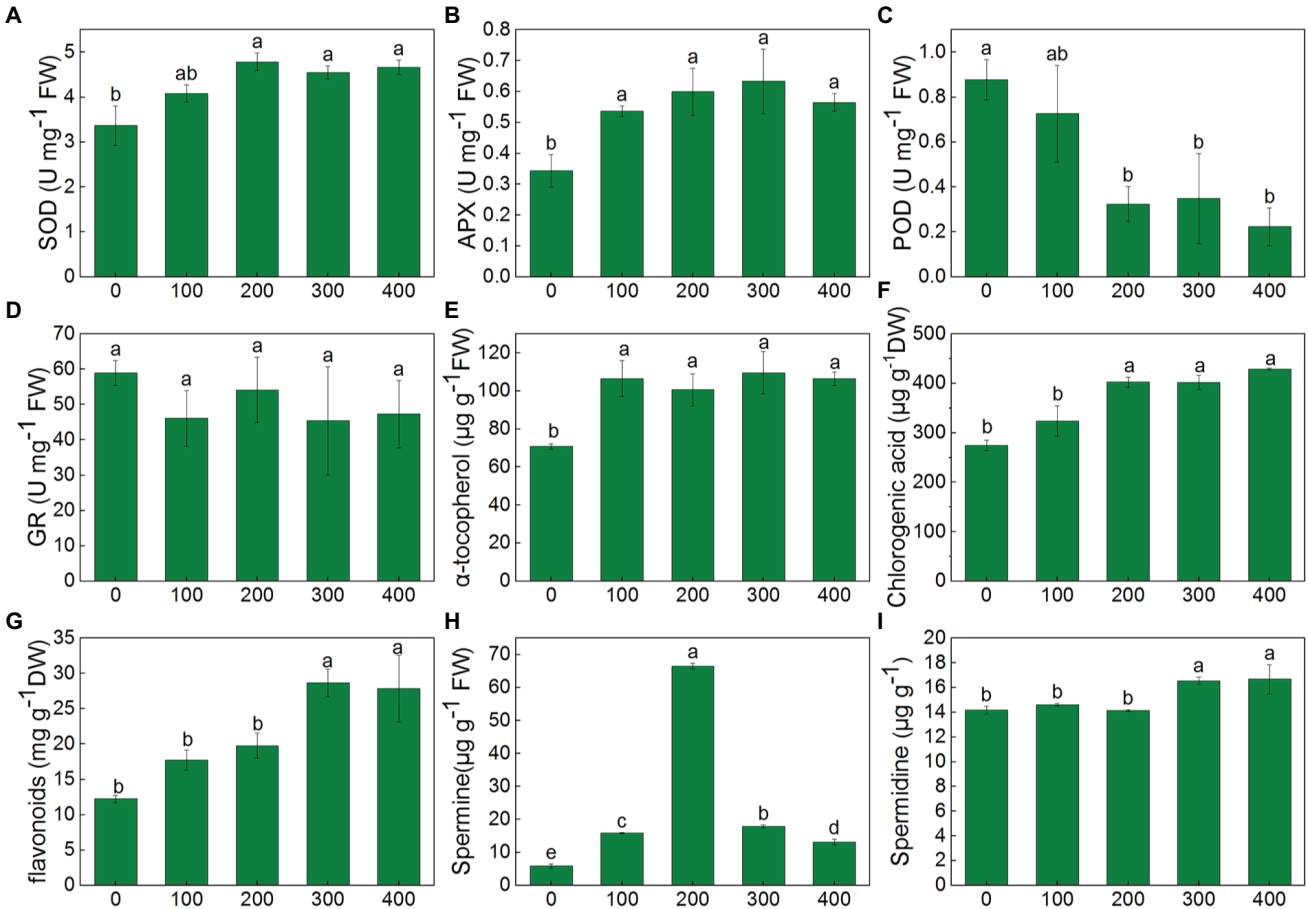
Changes of antioxidant enzymes and non-enzymatic antioxidants of *K. caspia* in response to NaCl treatment (100, 200, 300, and 400 mM). **(A)** SOD, **(B)** APX, **(C)** POD, **(D)** GR, **(E)** α-tocopherol, **(F)** chlorogenic acid, **(G)** flavonoids, **(H)** spermine, **(I)** spermidine Different letters indicate significant difference at *p* < 0.05.

The activity of antioxidant enzymes showed different trends under NaCl treatments. SOD activity remained unchanged in 100 mM NaCl treatment, but it increased significantly under treatment from 200 to 400 mM compared with control. SOD activity increased by 42.0, 35.1, and 38.4%, respectively, in 200, 300, and 400 mM treatments, and there was no significant difference between treatments (Figure 6A). APX activity increased by 56.3, 74.5, 84.4 and 64.5%, respectively, in the treatment from 100 to 400 mM NaCl compared with control. However, no significant differences were observed between treatments (Figure 6B). Compared to control, POD activity remained unchanged in 100 mM treatments but decreased sharply by 63.1, 60.3 and 74.6% in 200, 300, and 400 mM treatments (Figure 6C). GR activity remained unchanged in all treatments compared with control (Figure 6D).

In *K. caspia*, the content of α-tocopherol significantly increased under treatments as compared to control, and it increased by 29.8–50.4% in NaCl treatments, but there were no significant differences between treatments (Figure 6E). The content of chlorogenic and flavonoids were all increased with salinity. The content of chlorogenic significantly increased in 200, 300, and 400 mM NaCl treatments by 46.6, 46.2 and 56.0%, respectively (Figure 6F). The flavonoids were found to be increased significantly in 300 and 400 mM NaCl treated plants as compared to control, and increased by 1.4 and 1.3 folds, respectively (Figure 6G). Spermine content significantly increased under salinity, and it increased by 2.7, 11.4, 3.1 and 2.2 folds, respectively, under the NaCl treatment from 100 to 400 mM (Figure 6H). Spermidine content was maintained at the same level as controls under salinity of 100 and 200 mM NaCl treatment, but increased by 16.7 and 17.8% under 300 and 400 mM treatment, respectively (Figure 6I). Correlation analysis between anti-oxidative components and oxidative stress indicators showed that SOD had a negative correlation with O_2_^−^, and positive correlation with chlorogenic acid and flavonoids. H_2_O_2_ exhibited a negative correlation with α-tocopherol (Figure 4B).

In *K. caspia*, the levels of total glutathione (GSH + GSSG) increased with the increasing NaCl concentrations. From a position of no significant difference with 100 mM NaCl treatments, total glutathione levels were significantly higher than control in 200–400 mM NaCl treatments. The levels of total glutathione increased by 35.1, 39.7 and 58.4% in 200, 300, and 400 mM NaCl treatments, compared to control, respectively (Supplementary Figure S2A). Although the GSH level decreased in the treatments compared with the control, there was no significant difference between treatments (Supplementary Figure S2B). The level of GSSG increased with the increasing NaCl treatment, and it was 1.9, 2.4, 2.5, and 3.1 fold that of the control, respectively, under 100, 200, 300, and 400 mM NaCl treatments (Supplementary Figure S2C). The ratio of reduced and oxidized glutathione decreased with salinity as compared to control, but there was no difference between treatments (Supplementary Figure S2D).

The level of total ascorbate (AsA + DHA) remained unchanged in 100 mM NaCl treatment but was 1.5, 1.7, and 2.0 fold greater than control in 200, 300, and 400 mM NaCl treatments, respectively (Supplementary Figure S2E). The level of AsA showed no difference in 100 mM treatment as compared to control, but it increased by 47.6, 88.2 and 92.0%, respectively, in 200, 300, and 400 mM treatments (Supplementary Figure S2F). The trend of DHA content was the same as that of the total ascorbate and reduced ascorbate. The level of DHA in 100 mM treatment was the same as control, but it increased by 49.3, 67.3 and 101.0%, respectively, in 200, 300, and 400 mM NaCl treatments (Supplementary Figure S2G). The level of total ascorbate increased by 48.8, 72.8 and 106.0%, respectively, in NaCl treatments from 200 to 400 mM. The AsA/DHA ratio was maintained at the same level in control and NaCl treatments (Supplementary Figure S2H). These results showed that ascorbate-glutathione cycle was likely to aid in maintaining the cellular redox status in *K. caspia* under salinity.

### Expression level of transporters, antioxidant enzyme genes and genes related to antioxidant synthesis and signal pathway

**Figure 7:**
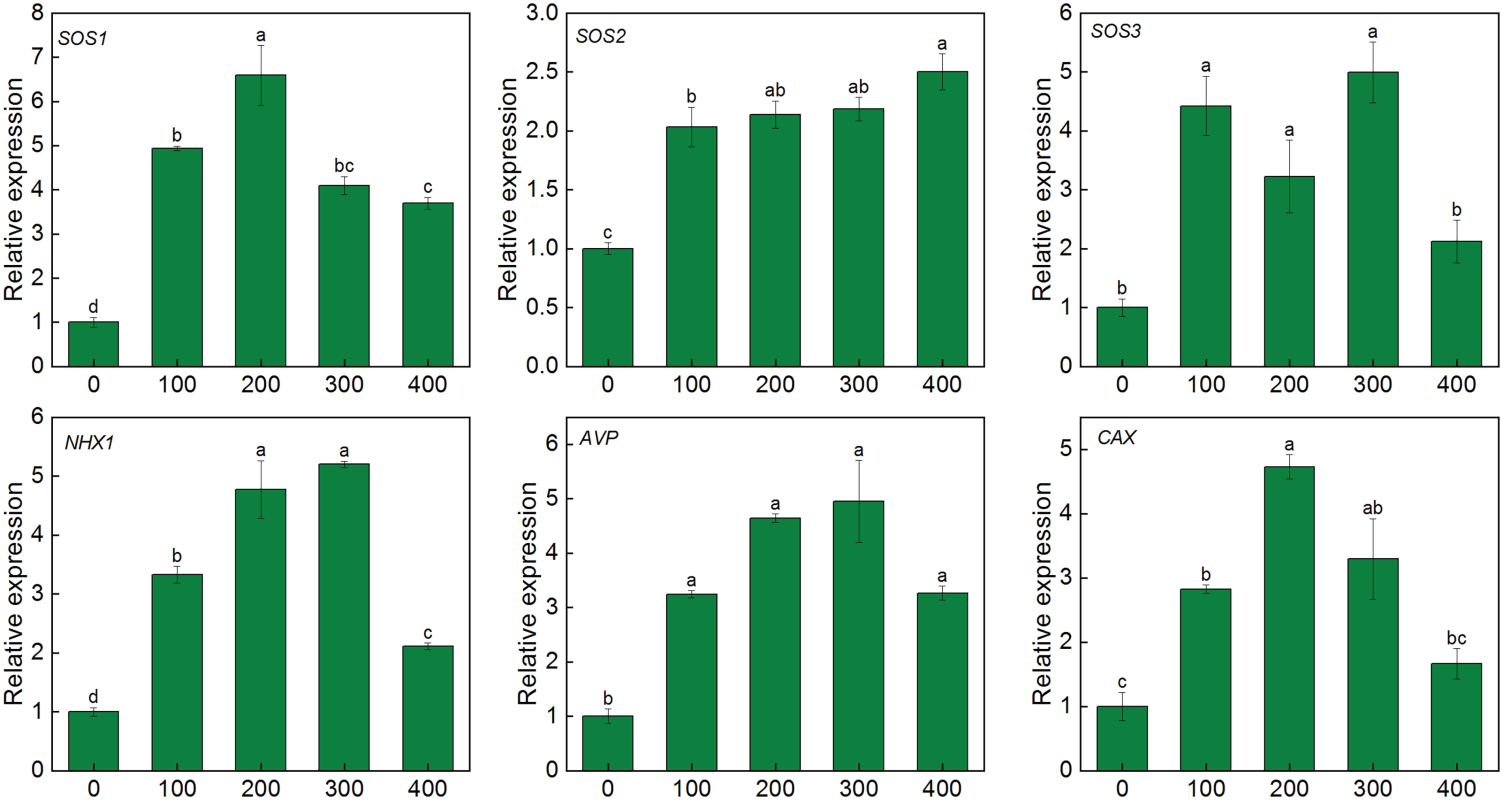
Expression level of ion transport-related genes in leaves of *K. caspia* under NaCl treatment (100, 200, 300, and 400 mM). salt overly sensitive 1/2/3(*SOS1/2/3*), tonoplast Na^+^/H^+^ antiporter1 (*NHX1*), vacuolar H^+^-pyrophosphatase pump (*AVP*), Ca^2+^/H^+^ exchanger (*CAX*).Different letters indicate significant difference at *p* < 0.05.

After 7 days of salt treatment, the expression of *SOS1/2/3* genes in leaves was significantly up-regulated. Compared with the control, the *SOS1* gene was up-regulated by 3.7–6.6 times, and the expression level was highest under 200 mM NaCl treatment. The expression of the *SOS2* gene increased with NaCl treatment concentration, and the expression level was up-regulated by 2.5 fold under 400 mM treatment. The *SOS3* gene was up-regulated by 2.1–5.0 fold under salinity. Similar patterns were also observed in *CAX* and *AVP* genes. The expression of *AVP* and *CAX* genes in leaves were up-regulated by 3.2–4.9 fold and 1.7–4.7 fold, respectively, under salinity (Figure 7).

Antioxidant enzyme genes and non-enzymatic antioxidant synthesis-related genes were all up-regulated in the leaves of *K. caspia* under salinity. The expression of *Cu/Zn SOD* was up-regulated by 1.6–2.6 fold, and it reached the highest value under 300 mM NaCl treatment. Similarly, the expression of *APX6* was also up-regulated under the 300 mM NaCl treatment. The expression of *HPT1* and *γ-TMT* was up-regulated by 1.2–1.4 and 1.4–2.1 fold, respectively, under salinity. The expression levels of *HCT* and *F3H* were up-regulated by 6.7 and 5.6 fold under 100 mM NaCl treatment, respectively (Supplementary Figure S3). The expression of *SAMS* increased significantly under salinity, and it increased by 5.1, 2.9, 3.8, and 3.1 fold under the treatments from 100 to 300 mM NaCl, respectively. The expression of *SMS* increased 2.5, 3.8, 2.9 and 2.5 fold under 100, 200, 300 and 400 mM NaCl treatment, respectively. MAPK cascade pathway related genes (*MEKK1， MKK2， MPK4/6*) were all increased under salinity. The expression of the four genes were up-regulated by 2.1–6.3, 1.2–6.1, 1.4–2.8 and 0.5–1.2 fold under NaCl treatments, respectively (Supplementary Figure S4). These results showed that salinity induced the up-regulated expression of kinase pathway, ions channels and antioxidant-related genes, which regulated the salt tolerance of *K. caspia.*

## Discussion

### Ion homeostasis and water maintenance under salt stress

Halophytes face the multiple challenges under salt stress, including ion toxicity and nutrient deficiencies (Acosta-Motos et al., 2017; Isayenkov and Maathuis, 2019). Halophytes can absorb Na^+^ and transport it to the aerial part, and finally localized Na^+^ into the vacuole to enhance tolerance to salt stress. In *K. caspia*, the concentration of Na^+^ in roots, stems, and leaves under salt treatments were all significantly higher than that in control, and the Na^+^ concentration in leaves was higher than that in roots and stems, which confirmed that *K. caspia* would uptake and accumulate more Na^+^ in leaves (Matinzadeh et al., 2019). Our results also demonstrate that a large number of Na^+^ was effectively sequestered into vacuoles, contributing to the osmotic adjustment of cytoplasm and maintaining the absorption of water and essential macronutrients (Rahman et al., 2019), then the relative water content of leaves was maintained at a steady level between control and NaCl treatments in *K. caspia*. In addition, scanning electron microscope results showed that the density of salt glands increased with the increase of NaCl treatment concentration. Especially in 300 and 400 mM NaCl treatments, the density of salt glands significantly increased as compared to control (Supplementary Figure S1), which indicated that salt secretion through salt glands is also an important strategy for *K. caspia* to cope with high salt stress.

The above results were further confirmed by the expression analysis of related genes of *K. caspia* under salinity. Salt overly sensitive (SOS) pathway, which comprises *SOS1*, *SOS2,* and *SOS3* genes, was an important pathway regulating Na^+^ homeostasis. *SOS1* was activated by the complex of *SOS2* and *SOS3* under salinity (Shah et al., 2021). Our previous research showed that *KcSOS1* functions in extruding Na^+^ from the cytoplasm of secretory cells to the exterior (Guo et al., 2020). In this study, the expression of *SOS1/2/3* were all up-regulated in the leaves of *K. caspia*, which further demonstrated that salinity activated the SOS pathway to expel excess Na^+^ from leaves through the salt glands. The *NHX1*, tonoplast Na^+^/H^+^ antiporter, functions in transporting Na^+^ to vacuoles (Rauf et al., 2014). *NHX1* was up-regulated both in leaves and roots of *K. caspia* under salinity, showing that Na^+^ was transferred into vacuole, and then accumulated higher Na^+^ in roots and leaves under salinity. The driving force of antiporters was created by H^+^-pyrophosphatase pumps, and is encoded by *AVP* gene. Gaxiola et al. (2001) reported that overexpression of *AVP* enhanced the tolerance to salinity by supplying H^+^ for *NHX1*-mediated Na^+^/H^+^ exchange, then contributing to the Na^+^ sequestration into the vacuoles. In this study, the *AVP* was up-regulated 4.9 fold, which confirmed that the *AVP* and *NHX1* combined to regulate the Na^+^ transportation to vacuoles. The regionalization of Na^+^ to vacuoles and the secretion of salt through salt glands jointly resisted the toxicity of Na^+^ to plants, thus ensuring the normal growth of plants.

Maintaining the homeostasis of Na^+^ and other ions is essential for plant metabolism. Potassium is the most abundant cation in plant cells and plays an essential role in enzyme activation, protein synthesis, membrane potential, ion homeostasis, and many other physiological processes (Chérel, 2004). There is a competitive relationship between Na^+^ and K^+^. Na^+^ enters cells through K^+^ channels and non-selective cation channels. The increase of Na^+^ is accompanied by the decrease in K^+^ (Rahman et al., 2019; Bueno et al., 2020). So, the concentration of K^+^ is 15 times that of Na^+^ in control, however, the concentration of K^+^ is only 0.2 times Na^+^ in leaves under salinity. Although K^+^ was significantly decreased in saline treatments compared with control in *K. caspia*, the concentration of K^+^ was maintained at a steady level among all treatments both in roots and leaves, and the K^+^/Na^+^ ratio also remained in a stable state in the roots, stems, and leaves. Further, *NHX1* was up-regulated in saline treatments. Bassil et al. (2011) reported that *NHX1* also mediates K^+^/H^+^ exchange to accumulate K^+^ in the vacuoles. Ca^2+^ and Mg^2+^ had no significant difference among salt treatments, and the concentration of both in leaves was significantly higher than that in stems and roots. The results indicated the Na^+^ sequestration in vacuoles reduced antagonistic effects to other ions, allowing other ions to be transported upward from roots. The trace elements Zn^2+^ and Fe^2+^ were all in a stable state among treatments. Our findings suggest that ion homeostasis is an important mechanism for the normal growth of *K. caspia* under high saline conditions.

### Osmotic adjustment of organic and inorganic substances under high salinity

Osmotic adjustment is an important physiological adaptation strategy against salt stress. Plants maintain water absorption by increasing osmolytes to reduce osmotic potential and maintain swelling pressure (Lu et al., 2021). In our study, the osmotic potential of *K. caspia* decreased significantly when exposed to salinity, and RWC was maintained at the same level between control and salinity. These results indicated that *K. caspia* has an effective osmotic adjustment mechanism. Low molecular compounds like proline and soluble sugars are considered important osmolytes (Parida et al., 2016). Our results showed that the content of proline increased significantly with the increase of salinity, but the content of proline was only 358 μg g^−1^, and the contribution rate was low. This suggested that proline was not the major osmotic substance in *K. caspia*, as also seen in *Halogeton glomeratus* (Lu et al., 2021). Soluble sugars increase significantly under the treatments of 300 and 400 mM NaCl, and their contribution to osmotic adjustment was higher than proline. However, Na^+^ accumulated significantly in *K. caspia*, and its contribution rate for osmotic adjustment was up to 57.6%, suggesting that Na^+^ may be the main osmolytes in *K. caspia*. In agreement with our results, inorganic substances were the major osmolytes have been reported in other halophytes like *Solanum chilense* and *Chenopodium quinoa* (Hariadi et al., 2010; Gharbi et al., 2017). Rana et al. (2015) suggested that the synthesis of organic substances is energy-consuming, and the energy consumption of Na^+^ transport is much lower than that of organic substances. This would be especially the case for halophytes, where the high concentration of Na^+^ in the soil is passively absorbed into plants. Thus, halophytes rely on Na^+^ as a “cheap osmoticum” to maintain cell turgor pressure (Zhao et al., 2020).

### Integrity of photosynthetic system II maintained the photosynthetic efficiency under high salinity

Photosynthetic pigments are essential determinants of photosynthetic capacity. Chlorophyll a and chlorophyll b are important components of photosynthetic system II. In our present study, the content of Chl a, Chl b, and the ratio of Chl a/b were all higher or equal to the control under salinity in *K. caspia*, which indicated that the chlorophyllase activity was unaffected by salinity. The integrity of chloroplasts in *K.caspia* enhanced the tolerance toward salt stress (Maršálová et al., 2016). These results contrast with the reports of the decreased chlorophyll under salinity in other halophytes (Al Hassan et al., 2017; Al-Shamsi et al., 2020). Chlorophyll a fluorescence parameters are regarded as important tools for analyzing the functionality of photosystems under salt stress. The F_v_/F_m_ is a sensitive indicator of photo-inhibition of PSII under stress (Chowaniec and Rola, 2022). In *K. caspia*, F_v_/F_m_ increased in salt treatments compared with control, indicating that salinity promoted the photosynthetic efficiency of PSII. Consistent with our result, F_v_/F_m_ increased under salinity in *Arthrocnemum macrostachyum* (Redondo-Gómez et al., 2010). PI_abs_ presents the performance index on an absorption basis, and ABS/CS_m_ represents the light energy absorbed per unit cross-sectional area (Kumar et al., 2020b). In *K. caspia*, PI_abs_ increased significantly compared with control, especially in 300 and 400 mM NaCl treatments. However ABS/CS_m_ was maintained at the same level as control. So our results showed that salinity did not affect the light-harvesting, and enhanced the light-absorbing performance of PSII. ET_0_/CS_m_ and RE_0_/CS_m_ represent the electron transfer efficiencies of PSII and PSI, respectively, and DI_0_/CS_m_ represents heat dissipation. In *K. caspia*, the electron transfer efficiency of PSII and PSI were all increased, while heat dissipation was decreased. The above results demonstrated that salinity did not cause damage to PSII, and further activated the activity of PSII by enhancing the efficiency of light-harvesting and electron transfer. This was further demonstrated by the increased net photosynthetic efficiency under salinity in *K. caspia*.

The net photosynthetic rate (Pn) increased under salinity in *K. caspia.* Similar results were also reported in halophyte *Atriplex portulacoides* and *Sarcocornia fruticose* (Redondo-Gómez et al., 2006). The improvement in the net photosynthetic rate may be attributed to the effective storage of Na^+^ in the vacuole and salt exclusion by the salt gland, which protected chloroplasts (Benzarti et al., 2014). This is also confirmed by the unaffected chlorophyll content and the increased F_v_/F_m_ in *K. caspia* under salt stress. In *K. caspia*, water use efficiency increased, stomatal conductance, and transpiration decreased under salinity. Similar results were also reported in halophyte *Achras sapots*(Rahman et al., 2019). Plants reduce stomatal conductance by closing stomata to reduce water loss due to transpiration. In halophytes, salt stress induces a decrease in stomatal conductance and transpiration rate, and thus improves water use efficiency as a strategy for water conservation (Panda et al., 2019).

### ROS scavenging through a combination of antioxidant enzymes and non-enzymatic antioxidants

It has been reported that salt stress induces excess ROS production, and ROS accumulation induces lipid oxidation, oxidative malfunction of protein and DNA (Chang et al., 2012). Furthermore, it also generates aldehyde substances like MDA that is considered an indicator of oxidative stress (Luna et al., 2000). In our study, O_2_^−^, H_2_O_2_ and MDA all exhibited no significant difference between control and NaCl treatments, and DAB and NBT staining also verified this result, indicating an effective antioxidative mechanism in *K. caspia*. Plants scavenge ROS and maintain ROS homeostasis through a regulation mechanism comprising antioxidant enzymes and non-enzymatic antioxidants (Al Kharusi et al., 2019). SOD is considered the primary enzyme to defend against oxidative stress and catalyzes the dissimulation of O_2_^−^ to H_2_O_2_ and O_2_ (Bose et al., 2014). In *K. caspia*, SOD increased significantly under high salinity (200-400 mM). Correlation analysis showed that SOD had a negative correlation with O_2_^−^, and the expression level of *Cu/Zn SOD* gene increased with the increase of NaCl treatment, indicating that salinity induced the expression of *Zn-Cu SOD* of *K. caspia*, then the increased SOD activity regulated the proper level of O_2_^−^. So, O_2_^−^ was maintained at a steady state when exposed to salt stress. APX, POD, and GR were key enzymes scavenging H_2_O_2_ (Shigeoka et al., 2002). In *K.caspia*, APX activity was significantly increased, and the expression level of *KcAPX6* also increased when exposed to salinity, demonstrating that APX plays an important role in H_2_O_2_ scavenging. POD activity decreased in salinity, but GR appeared stable under salinity. The activity of antioxidant enzymes are different in other halophytes when exposed to salinity (Yu et al., 2011). Our results showed that SOD and APX were the key antioxidative enzymes participating in ROS scavenging in *K. caspia*.

Non-enzymatic antioxidants play an important role in scavenging ROS and avoiding antioxidant damage. In our study, non-enzymatic antioxidants such as ascorbic, glutathione, α-tocopherol, chlorogenic acid, and flavonoids were measured. Ascorbate and glutathione scavenge H_2_O_2_ and maintain cellular redox status through the ascorbate-glutathione cycle (Foyer and Noctor, 2005). In *K. caspia*, the level of AsA, DHA, and AsA + DHA were all increased significantly when exposed to salinity from 200 to 400 mM NaCl. AsA has a vital role in maintaining APX activity for detoxication of H_2_O_2_ (Rangani et al., 2018), this is verified by increasing APX activity under saline treatment in *K. caspia*. The higher AsA level and APX activity in *K. caspia* contributed to their increased antioxidant capacity and tolerance to salinity. Furthermore, AsA/DHA ratio was maintained at the same level in NaCl treated plants of *K. caspia*. The steady ratio of AsA/DHA maintains the appropriate redox of the cell to mitigate the salt-induced oxidative stress. Unlike ascorbate, reduced glutathione(GSH) levels were significantly decreased as compared to control in NaCl treatments, but the level of GSH remained in a stable state under salinity. The lower GSH signifies the high rate of conversation of GSH into the production of GSSG, and the higher GSSG level helps maintain a steady level of GR activity (Panda et al., 2021). Although the GSH/GSSG decreased in NaCl treatment compared with control, the ratio of GSH/GSSG was maintained at the same level among NaCl treatments. Our results suggested that GSH and AsA, through the ascorbate-glutathione cycle, maintained the cellular redox status in *K. caspia* under salinity.

α-tocopherol is a lipid-soluble antioxidant best known for its ability to scavenge ROS. In *K. caspia*, the level of α-tocopherol significantly increased under salinity. Furthermore, expression of the genes *Kcγ-TMT* and *KcHPT1*, the key synthase genes for α-tocopherol, were significantly up-regulated under salt treatment. Studies have shown that overexpression of *Kcγ-TMT* and *KcHPT1* can increase the content of tocopherols in plants, thereby enhancing the salt tolerance of plants (Jin and Daniell, 2014). α-tocopherolis synthesized in the chloroplast, and it cooperates with ascorbic and glutathione to effectively scavenge and quench various free radicals, then maintain the homeostasis of ROS. This can helps protect photosystem II from damage caused by stress (Czarnocka and Karpiński, 2018; Kumar et al., 2020a). In *K. caspia*, α-tocopherol may maintain the integrity of the photosystem II by scavenging ROS, thereby improving the salt tolerance of *K. caspia.*

Chlorogenic acid and flavonoid belong to polyphenols, which are natural non-enzymatic antioxidants in plants (Sullivan and Michael, 2009). In *K. caspia*, the content of chlorogenic and flovanoids increased significantly under salinity from 200 to 400 mM and 300–400 mM respectively, and chlorogenic acid synthase *KcHCT* and flavonoid synthase *KcF3H* were all up-regulated under salinity. Other researchers have reported similar results. For example, salinity induced high expression of *HCT* in *Hibiscus cannabinus* (Chowdhury et al., 2012). Polyphenols such as chlorogenic acid and flavonoids accumulated in lettuces under salt stress (Sgherri et al., 2017). In many medical plants, phenolic compounds contribute to the trapping of free radicals (Bistgani et al., 2019). The increase of polyphenols enhanced the antioxidant activity, to protecting the plant from the damage of lipid peroxidation as indicated by the MDA levels. In line with this, the exogenous application of chlorogenic acid can alleviate the toxic effect of salt stress on plants (Zhang et al., 2021). So, in *K. caspia*, high salinity induced the production of chlorogenic acid and flavonoids, conferring the tolerance of *K. caspia* to salinity.

Polyamines (spermine, spermidine and putrescine) are aliphatic polycations that participate in the response to environmental stress (Liu et al., 2020). The accumulation of spermine was considered an important indicator of salt tolerance in plants (Simon-Sarkadi et al., 2007). Exogenous addition of polyamines or overexpression of polyamine synthesis genes could improve plant tolerance to abiotic stress (Zhang et al., 2015; He et al., 2019). Spermine also acts as signaling molecules to activate antioxidant enzyme activity or directly scavenge reactive oxygen species to regulate the homeostasis of ROS in plants (Tanou et al., 2014; Seifi and Shelp, 2019). In our experiment, the content of spermine was significantly increased under salinity, and the key associated biosynthetic enzymes such as *KcSMS* and *KcSAMS* were all up-regulated. This indicated that salinity induced the accumulation of polyamines in *K. caspia,* to enhanced the tolerance to salt stress. Elevated spermine is also an important osmoprotectant against salt stress (Paul and Roychoudhury, 2016). Furthermore, spermine may be involved in the excretion process and regulate ions fluxes through salt gland (Ben Hassine et al., 2009). Malliarakis et al. (2015) also reported that polyamine could restore PSII efficiency by interacting with PSII thylakoid proteins. So, elevated could regulate plant tolerance to salt stress by multiple regulatory pathways.

### Signal transduction under salt stress in *Karelinia Caspia*

Plants produced a large amount of ROS when exposed to abiotic stress which can inhibit plant development or lead to plant death (Anjum et al., 2015). However, low level of ROS also has been considered as the key players of stress signaling in plants. It has been reported that H_2_O_2_ acted as a signaling molecular activated protein kinases or transcription factors, thereby activating the expression of related genes to resist abiotic stress (Hossain et al., 2015; Mansoor et al., 2022). In our experiment, the content of H_2_O_2_ in *K. caspia* was in a steady state at a low level under salinity, which indicated that H_2_O_2_ may played important role as a signaling molecule in response to salt stress.

**Figure 8:**
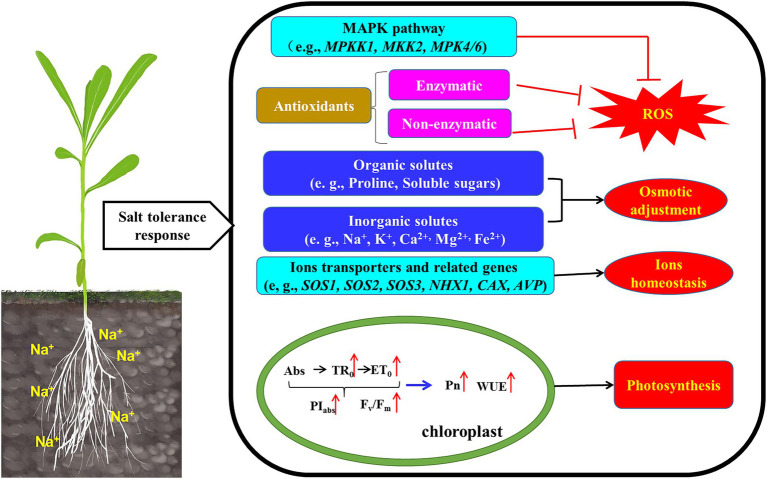
Schematic model illustrated that the mechanism of *K. caspia* resisted salt stress by regulating ROS homeostasis, osmotic balance, ion homeostasis, and photosynthesis. (1) The expression of MAPK pathway genes (*MEKK1-MKK2-MPK4/6*) were up-regulated, which crosstalk to ROS scavenging. (2) Antioxidant enzyme (SOD, APX) activity increased to reduce the excess ROS. The accumulation of α-tocopherol, chlorogenic acid, flavonoids and polyamines also scavenge ROS. Antioxidant enzyme and non-enzymatic antioxidants combined to maintain ROS homeostasis. (3) Accumulating organic solutes (soluble sugars, proline, polyamines) contributed to osmotic adjustment. (4) The up-regulated *SOS2* activated the transporter activity of *SOS1* and *CAX* in the plasma membrane, and extruded Na^+^ into the apoplast and sequestrate Ca^2+^ into the vacuoles, and the *NHX1* was also activated to sequestrate Na^+^ into the vacuoles. The accumulation of Na^+^ in vacuoles contributed to osmotic adjustment. (5) Increased chlorophyll fluorescence parameters enhanced photosynthesis efficiency and water use efficiency, then maintained the normal growth of *K. caspia*.

The mitogen-activated protein kinase (MAPK) cascade is a universal signal transduction module and involved in stress-related pathways (Singh and Jwa, 2013), and *MEKK1, MKK2, MPK4/6* was activated by salt stress. In *K. caspia*, *KcMEKK1*, *KcMKK2*, *KcMPK4* and *KcMPK6* had the same expression patterns under different concentration of salt stress, and they were all up-regulated by salinity. It is reported that *MPK4* and *MPK6* has an important role in salt stress signaling (Ichimura et al., 2000). Völz et al. (2022) showed that ROS homeostasis was mediated by *MPK4*, and *mpk4* mutant showed accumulation of hydrogen peroxide (Völz et al., 2018). *MPK4* may also be involved in photosynthetic electron transport and chloroplast ROS metabolism. Yu et al. (2010) reported that *MPK6* phosphorylates *SOS1*, and further participated the regulation of Na^+^ homeostasis. Activation of the protein kinase pathway enhanced plant tolerance to salt stress by regulating downstream defense responses. Taken together our data suggested roles for antioxidants, osmolytes, ion homeostasis and signal crosstalk in *K. caspia* subjected to salt stress. To ease comprehension these are illustrated schematically in Figure 8.

## Conclusion

This study described mechanisms of salt tolerance in halophyte *K. caspia.* The results showed that the optimal growth condition is 100 mM NaCl treatment. The content of Na^+^ in roots, stems and leaves of *K. caspia* increased significantly under salinity. Na^+^ was compartmentalized into vacuoles and acted as the main osmotic adjustment substance to maintain the water balance of *K. caspia* under salinity. The excessive Na^+^ was excreted through the salt glands, and the salt stress increases the number of salt glands. The antioxidant enzymes (SOD, APX) and non-enzymatic antioxidants (α-tocopherol, chlorogenic acid, flavonoids, polyamines) combined to regulate the ROS homeostasis. So, the contents of H_2_O_2_, O_2_-, and MDA can be maintained at appropriate levels under salt treatments. Salinity improved the performance of PSII. Meanwhile, the net photosynthetic rate and water use efficiency were all increased. Therefore, *K. caspia* can grow normally under high saline stress, through the combined action of ions regulation mechanism and, antioxidant defense systems, the higher tolerance index make *K. caspia* a suitable candidate for exploring saline-alkali tolerant resources and the restoration of saline-alkali land.

## Data availability statement

The original contributions presented in the study are included in the article/Supplementary materials; further inquiries can be directed to the corresponding authors.

## Author contributions

QG and JW designed the research. CL, QW, XH, CZ, and ZC performed the experiments. CL and QG analyzed the data. CL wrote the manuscript. QG, JW, and LM edited and revised the manuscript. All authors contributed to the article and approved the submitted version.

## Funding

This study was funded by the Young Project of Beijing Academy of Agriculture and Forestry Sciences (QNJJ202221), Innovative Project of Beijing Academy of Agriculture and Forestry Sciences (KJCX20220407), National Natural Science Foundation of China (31601991), Biotechnology and Biological Sciences Research Council (BBSRC, United Kingdom) “A China–UK consortium to reduce environmental pollution with novel grass varieties” (BB/M027945/1).

## Conflict of interest

The authors declare that the research was conducted in the absence of any commercial or financial relationships that could be construed as a potential conflict of interest.

## Publisher’s note

All claims expressed in this article are solely those of the authors and do not necessarily represent those of their affiliated organizations, or those of the publisher, the editors and the reviewers. Any product that may be evaluated in this article, or claim that may be made by its manufacturer, is not guaranteed or endorsed by the publisher.
