# Supplementary material for: ROS scavenging and ion homeostasis is required for the adaptation of halophyte Karelinia caspia to high salinity
Source: Front Plant Sci. 2022 Oct 3;13:979956. doi: 10.3389/fpls.2022.979956 (PMC9574326; doi:10.3389/fpls.2022.979956)
Supplement: Supplementary file 1 [file Data_Sheet_1.docx]

Supplementary Material

**Table S1.** Primer sequences used in the experiments

| Primer | Sequence (5’-3’) |
| --- | --- |
| *KcActin*-F | 5’-CTTGCGTATGTGGCTCTTGACT-3’ |
| *KcActin*-R | 5’-TGGAACAAAACCTCTGGACAAC-3’ |
| *KcSOS1*-F | 5’-TGTGTGGTCTGGTTTGAGGGGT-3’ |
| *KcSOS1-R* | 5’-TCTGATTGGGTATGAGGGTGGA-3’ |
| *KcSOS2*-F | 5’-GAAGCTGGAACCTGTGGCGAAG-3’ |
| *KcSOS2*-R | 5’-CATGTCCCTGGAGCCTCAAACTG-3’ |
| *KcSOS3*-F | 5’-TCCACATTCTCAGTCGCTGTAAGTG-3’ |
| *KcSOS3*-R | 5’-ACACCGTCGTTGATGATGGAGTTG-3’ |
| *KcNHX1*-F | 5’-GGGTGTGGTAAATGATGCAACAT-3’ |
| *KcNHX1*-R | 5’-CAACAAAAACTCCCAACAAGGTG-3’ |
| *KcCAX*-F | 5’-AACTTTGGACTCACCGCCACATC-3’ |
| *KcCAX*-R | 5’-CCTAGCACTGGGGTTTCATCTTCAC-3’ |
| *KcAVP*-F | 5’-GAGACGGCAGTTCAACACCATCC-3’ |
| *KcAVP*-R | 5’-AACAAGAGCACCAGGAGGAATCATC-3’ |
| *KcCu/Zn SOD*-F | 5’-GTCACAGTTGGCGAAGACGGTAC-3’ |
| *KcCu/Zn SOD-R* | 5’-ATCAGGGTCAGCATGGACAACAAC-3’ |
| *KcAPX6*-F | 5’- TTTAGGGAGGTCCCGACCAGAAC -3’ |
| *KcAPX6-*R | 5’-GCCACTTCACTGTCCAAGACTGTC -3’ |
| *KcHPT1-*F | 5’-GGTTGGATGGATTGTTGGTTCATGG-3’ |
| *KcHPT1-*R | 5’-ATGCACATTGCTGCCACGAGAG-3’ |
| *Kcγ-TMT*-F | 5’-TCACGGCTTCTACGACCCTGAC-3’ |
| *Kc γ-TMT-*R | 5’-AGGAACAGAGGCGAAGAGGAGTG-3’ |
| *KcHCT-*F | 5’-GGAGGAGTGGGTCTTGGTTGTGG-3’ |
| *KcHCT-*R | 5’-AATGAAAGGTGGAATGGCTACGG-3’ |
| *KcF3H-*F | 5’-CAAAGTGTCCTAAGCCCGACCTAAC-3’ |
| *KcF3H-*R | 5’-TGTGATCCATGTCTTGCCACCATC-3’ |
| *KcSAMS-F*  *KcSAMS-R*  *KcSMS-F*  *KcSMS-R*  *KcMEKK1-F*  *KcMEKK1-R*  *KcMKK2-F*  *KcMKK2-R*  *KcMPK4-F*  *KcMPK4-R*  *KcMPK6-F*  *KcMPK6-R* | 5’-AAGCTCAACCCCACCCTCATACTAG-3’  5’-ACTCCAGCGGAAAACACATGCC-3’  5’-TGGCAGAGAGTATGTGGCTCCAC-3’  5’-GACGCTGGTCCATGCATAATGAATG-3’  5’-GTGATTTTCTTGGAAGTGGGTCGTT-3’  5’-TGCTTTCCTTTGGTCCCTTGGT-3’  5’-GCCAATCTGATACTCCGCCACTT-3’  5’-CACCGTTCCCTTTTCCAACTACTTTA-3’  5’-AGAGGAGCTTACGGAATTGTCTGTGC-3’  5’-GGTCCGTTTGGCATCTATTCTGTTGT-3’  5’-CGCAAGCCCTTATTTCCTGGTAG-3’  5’-GAAACCCCAATTCAGCTTCTGATGG-3’ |


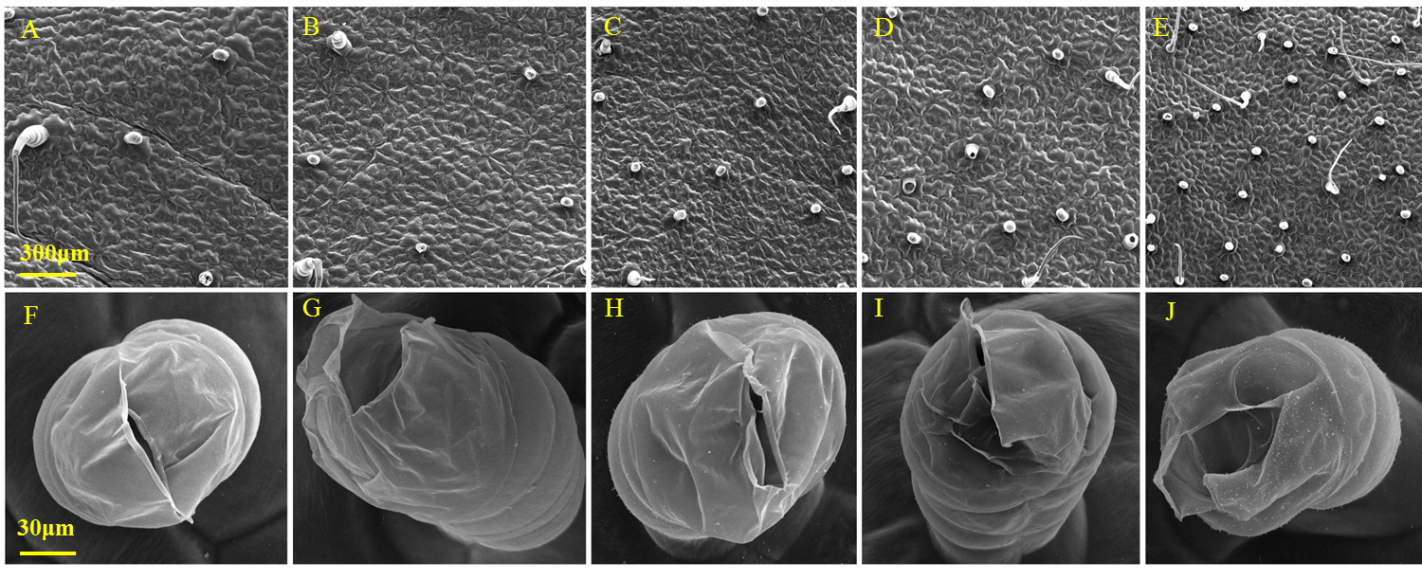


**Figure S1** Salt glands of *K. caspia* under different NaCl treatments (A-F) distribution of salt glands on leaf surface; (F-J) salt glands; (A,F) CK, (B,G) 100 mM NaCl, (C,H) 200 mM NaCl, (D,I) 300 mM NaCl, (F,J) 400 mM NaCl

**Figure S2** Changes in the glutathione and ascorbate content of *K. caspia* in response to salinity. (A) total glutathione (GSH+GSSG), (B) reduced glutathione (GSH), (C) oxidized glutathione (GSSG), (D) GSH/GSSG ratio. (E) reduced ascorbate(AsA), (F) oxidized ascorbate (DHA), (G) total ascorbate (AsA+DHA), (H) AsA/DHA ratio.

**Figure S3** Expression level of antioxidant enzyme genes and antioxidant synthesis related genes in leaves of *K.caspia*. (A) *Cu/Zn SOD*; (B) ascorbate peroxidase6 (*APX6*); (C) homogentisate phytyltransferase (*HPT1*); (D) γ-tocopherol methyltransferase (*γ-TMT*); (E) hydroxycinnamoyl transferase (*HCT*); (F) flavanone 3-hydroxylase (*F3H*)

**Figure S4** Expression level of MAPK pathway related genes in leaves of *K. caspia*. (A) mitogen-activated protein kinase kinase kinase 1(*MEKK1*); (B) mitogen-activated protein kinase kinase2 (*MKK2*); (3) mitogen-activated protein kinase4 (*MPK4*); (4) mitogen-activated protein kinase6 (*MPK6*)
